# Analyzing influencing factors to scale up agroforestry systems in Colombia: A comparative *ex-ante* assessment of cacao farming and cattle ranching in two regions

**DOI:** 10.1007/s10457-022-00730-1

**Published:** 2022-01-18

**Authors:** Tatiana Rodríguez, Michelle Bonatti, Katharina Löhr, Marcos Lana, Martha Del Río, Stefan Sieber

**Affiliations:** 1 Leibniz Centre for Agricultural Landscape Research (ZALF), Sustainable Land Use in Developing Countries (SusLAND), Müncheberg, Germany; 2 Agricultural Economics, Humboldt-Universität zu Berlin, Berlin, Germany; 3 Urban Plant Ecophysiology, Humboldt-Universität zu Berlin, Berlin, Germany; 4 Crop Production Ecology, Swedish University of Agricultural Sciences, Uppsala, Sweden

**Keywords:** Scaling-up, Impact assessment, Land management, Sustainable Cacao Production, Silvopastoral systems

## Abstract

Agroforestry systems (AFS) are proved to enhance sustainable land management. Thus, there is increasing demand for effective ways to scale up AFS so that more people can benefit. Consequently, this study assesses the scaling-up potential of agroforestry systems (AFS) using cacao farming and cattle ranching in Caquetá and Cesar, Colombia, as examples. An *ex-ante* assessment using the ScalA tool is conducted through interviews with AFS experts from institutions promoting AFS. Using a comparative approach, results reveal that AFS have different scaling-up potential depending on the type of farming system and location characteristics. In our case, it is slightly higher for cacao farming than for cattle ranching in both regions and it is higher in Caquetá than in Cesar for both systems. Factors hindering the scaling-up potential for both regions are economic conditions at the local and regional levels since there is a lack of stable and differentiated markets to absorb AFS products. In contrast, the scaling-up potential in both regions is increased by the factors related to the capacity of the organizations that promote AFS and the attitudes of local communities toward them. The study generates information about factors that may hinder or foster AFS scaling-up, including not just the capacities and mechanisms to promote them but also the enabling conditions. This contributes to prioritizing AFS interventions and better allocating their resources to increase their chances of successful scaling-up.

**Supplementary Information:**

The online version contains supplementary material available at 10.1007/s10457-022-00730-1.

## Introduction

Agroforestry systems (AFS) are sustainable land management strategies that deliberately integrate woody perennials, herbaceous plants, livestock, and people, and their interactions with one another in farming and forest systems (Sinclair 1999; Zomer et al. 2009; Nair and Garrity 2012). These are traditionally employed by smallholder farmers throughout the tropics and promoted as a sustainable livelihood alternative by land-use managers and multilateral agencies through technical services, education, research, laws, and institutions (Zomer et al. 2009; Somarriba et al. 2012; Reij and Garrity 2016). AFS are considered key to reducing deforestation, facilitating ecosystem conservation, and mitigating and enhancing resilience to climate change (Pagiola et al. 2010; Lasco et al. 2014; Jacobi et al. 2015; Waldron et al. 2017; Nyong et al. 2020).

This is particularly important for a tropical emerging economy like Colombia, which has around 50% of its territory forested (593,270 km^2^), but is dealing with alarming deforestation rates. Approximately 1972 km^2^ were deforested in 2018 (SMByC, 2018). AFS are appropriate for 16.3% of Colombian land, not just balancing sustainable production against natural resource depletion (IGAC 2017) but also offering socioeconomic and environmental benefits at the farm level (Tapasco et al. 2019). However, AFS are not fully established on potential agricultural land and their contributions remain undeveloped, just as in the rest of the world (Das et al. 2021; Akamani and Holzmueller 2017; Montes-Londoño 2017; Guteta and Abegaz 2016; Jerneck and Olsson 2014). An adequate and careful process of AFS scaling-up could help to reduce pressures on natural forests, thus decreasing deforestation (Lerner et al. 2017; Castro-Nunez et al. 2021).

Cacao agroforestry systems (CAFS) could be considered a prevalent AFS type in Colombia since cacao crops are typically established and managed under shade by smallholder farmers with differing production practices dependent upon climate, soils, and household needs. CAFS tend to include banana plants, fruit trees, and shade trees mainly with timber species (Abbott et al. 2018), characteristics in line with CAFS definitions, which indicate they are complex multi-species cropping systems where cacao trees are associated with other permanent or temporary crops and with woody tree species (Cerda et al. 2014; Jagoret et al. 2014). However, CAFS still need better management since cacao yields are low and the potential benefits of harvesting timber are limited (Abbott et al. 2018).

On the other hand, silvopastoral systems (SPS) are an alternative for sustainably managing the already established cattle ranches in Colombia (Zuluaga and Etter 2018; Jose and Dollinger 2019), which traditionally have high environmental impact and low productivity, but are relevant in socioeconomic terms as they are deeply rooted in the culture and their derived products are still in high demand (Mauricio et al. 2018). SPS in Colombia combine fodder plants, like grasses and leguminous herbs, with shrubs and trees on the same unit of land, for mainly animal nutrition and comfort (Calle et al. 2013; Jose et al. 2019). Some examples include scattered trees in pasturelands, living fences, mixed fodder banks, and intensive silvopastoral systems (Calle et al. 2013).

As multiple AFS exist and their implementation is highly context dependent, there is no single formula to successfully scale up them (Franzel et al. 2004; Jerneck and Olsson 2014). Following the definition of Hartmann and Linn (2008), scaling-up refers to expanding, adapting, and sustaining successful policies, programs, or projects in different places and over time. However, it is not just about impact, scale, and sustainability, but it also involves a multidimensional process of change and adaptation that can be achieved by influencing the political processes, or by involving and working with other stakeholders and institutions.

Research shows the crucial role of service delivery mechanisms for AFS scaling-up, including participatory and farmer-centered research and extension approaches that facilitate cooperation between farmers, extensionists, and researchers. These approaches enable co-learning among AFS actors, helping to identify the most important knowledge gaps, thus prioritizing extension services and communication strategies, while a variety of technical options could be developed with fine-scale variations that adequately integrate trees, crops, and/or livestock within the farming systems considering the local circumstances (Franzel et al. 2004; Calle et al. 2013; Coe et al. 2014; Guteta and Abegaz 2016; Reij and Garrity 2016; Baig et al. 2020). An adequate institutional context is also relevant to scale up AFS, which implies not only the building of local institutional capacities or establishment of strategic partnerships (Franzel et al. 2004; Calle et al., 2013; Macke et al. 2021) but also institutional buy-in to influence the public policy agenda (Calle et al. 2013; Chavan et al. 2015; Reij and Garrity 2016). In addition, enabling economic and market conditions are needed to effectively scale up AFS (Baig et al. 2020), potentially including the development of certification programs or marketing strategies that help farmers to eliminate middle men or obtain premium prices (Guteta and Abegaz 2016; Reij and Garrity 2016; Pandit et al. 2019).

Existing studies typically focus on single farming systems and regions. However, a comparative approach will not just show the context specificity of AFS scaling-up but also identify factors influencing this process across farming systems and regions, thus being relevant to diverse stakeholders and contexts. Therefore, this study seeks to comparatively assess the hindering and fostering factors affecting the scaling-up potential of SPS and CAFS across two regions of Colombia (Caquetá and Cesar). The study is guided by one research question: what are the hindering and fostering factors—including their interrelationships—for SPS and CAFS scaling-up in Caquetá and Cesar? Our hypothesis, based on the current literature, is that economic conditions might be the greatest hindrance to scaling-up SPS and CAFS in both regions. However, they are interacting with the social, institutional, and environmental conditions of each region that might also hamper this process.

## Methodology

### Study regions: Caquetá and Cesar departments of Colombia

The scaling-up potential of SPS and CAFS is investigated in two contrasting regions in Colombia, namely Caquetá and Cesar. Both are affected by high deforestation rates but in different ways due to their differing social, economic, and environmental conditions. Caquetá has abundant natural wealth, including water, due to its geographical location in the Amazonian region and the environmental regulation restricting land-based production activities (SIPRA 2019). However, it is at risk due to high deforestation rates (Castro-Nunez et al. 2021), which have increased since the peace agreement was signed in 2016 (Enciso et al. 2018). One main driver of deforestation is the expansion of pasturelands, reflecting that cattle ranching is the most representative economic activity there (Landholm et al. 2019). In contrast, Cesar, on the Caribbean plain, also hosts a high diversity of landscapes ranging from the mountain ranges of the Sierra Nevada de Santa Martha and the Serranía del Perijá to the valleys of the rivers Magdalena and Cesar (IGAC 2017). Palm oil cultivation and cattle ranching are the most relevant agricultural activities. Nevertheless, land occupation without environmental planning has brought about an acute loss of tropical dry forest and increased soil degradation. This situation has been exacerbated by the effects of climate change, which has led also to longer droughts and water shortages (ADR and FAO 2019).

### Ex-ante assessment tool

An *ex-ante* assessment was conducted using the questionnaire of the Scaling-up Assessment Tool (ScalA). ScalA is designed to systematically assess (*ex-ante*) the degree of sustainability as well as the scaling-up potential of agricultural interventions (Crewett et al. 2005). The tool has been applied to studies assessing the scaling-up potential of a set of sustainable agricultural practices in a region (Jha et al. 2020; Sieber et al. 2015) or a sustainable strategy among a set of regions (Bonatti et al. 2017). The results of this ex-ante assessment could be useful to tailor sustainable AFS scaling-up interventions to the context, implement them through better planned strategies, and prevent negative impacts in local communities (Pope et al. 2013; Schindler et al. 2015).

ScalA only prompts for the scaling-up potential assessment if the agricultural intervention is perceived as sustainable. When this is verified through a checklist of 17 sustainability indicators, the scaling-up potential assessment is conducted. For this purpose, the tool defines 59 scaling‐up factors divided into 7 categories including AFS attributes, capacities of implementing organizations, attributes of AFS scaling-up strategies, national-level political/institutional framework, local institutional setting, local/regional economic conditions, and community attitudes toward AFS. The users score the compliance of these factors using a Likert-type scale with 0 if the factor is not met or not met at all, 1 if it is not completely met or there are some other limitations, and 2 if it is very much or very well met. The tool also weighs the importance of associated scaling-up factors by assessing the relevance of 11 financial, human, institutional, and agricultural inputs requirements for implementation using a scoring system between 0 (not relevant) and 3 (significant). For example, a high financial capital requirement leads to a high relevance of the factor related to farmers' affordability to implement AFS. As a result, the user obtains a percentage that indicates a deviation of a current scaling-up situation from an optimal scaling-up situation where a predefined set of factors are fulfilled. Thus, a lower percentage denotes a higher chance for AFS scaling-up (Crewett et al. 2005; ZALF 2020).

### Data collection

As scaling-up implies coordinated processes between stakeholders to expand and sustain AFS vertically (Hartmann and Linn, 2008), we conducted the ex-ante assessment of SPS and CAFS scaling-up potential with diverse stakeholders from Cesar and Caquetá using the ScalA questionnaire. It was applied 18 times through 16 interviews and 2 focus groups between February and May 2020, involving a total of 27 stakeholders (Table 1). These respondents were selected in two stages. First, we created a list of governmental and non-governmental institutions working on cacao farming and cattle ranching in both regions through a web search. Then, we chose experts to be interviewed from the list of institutions using three criteria: (1) their thorough knowledge about SPS or CAFS; (2) their experience with cacao farming or cattle ranching in Cesar or Caquetá; and (3) their experience implementing SPS or CAFS in one of the regions. The sample was diversified in terms of respondents’ positions (regional heads, project coordinators, researchers, and technical assistants), institutional missions (extension, research, and education), and scale (national, regional, and local). Farmer perspectives were included in the sample since four technical advisors interviewed were farmers.

**Table 1:** Number and nature of questionnaires conducted, stakeholders interviewed, and institutions involved in assessing scaling-up potential of SPS/CAFS in the two regions

	Cesar		Caquetá	
	SPS	CAFS	SPS	CAFS
Number of questionnaires and their method of application	(5) Individual interviews (1) Focus group	(5) Individual interviews	(3) Individual interviews	(3) Individual interviews (1) Focus group
Number and role of the stakeholders interviewed	(2) Project coordinators (11) Researchers (1) Technical advisor	(1) Regional head (1) Researcher (3) Technical advisors	(2) Project coordinators (1) Researcher	(1) Regional head (2) Researchers (2) Technical advisors
Number and type of the institutions involved	(1) NGO (1) Locally based rural extension institution (1) Regional university (1) Livestock extension institution (1) Agricultural research center	(1) NGO (1) Locally based rural extension institution (1) National federation for cacao producers (1) Agricultural research institution (1) Cacao trading company	(2) NGOs (1) Agricultural research institution	(1) National federation for cacao producers (2) Agricultural research institutions

After conducting with respondents the sustainability assessment, they were not only asked to score the 59 scaling-up factors and 11 implementation requirements but also encouraged to comment on their scores. Scoring during the focus groups was done by consensus. Each interview lasted around 120 min and was conducted in Spanish face-to-face, by video call, or by phone call. Initially, all interviews were planned face-to-face; however, mobility restrictions due to the Covid-19 pandemic required methodological adaptions. All interviews were recorded and transcribed. To increase study reliability and validity, we also used secondary information (including papers, reports, and websites of organizations) to complement the empirical data.

### Data analysis

Respondents’ verbal comments regarding individual scaling-up factors and resource requirements were analyzed via qualitative content analysis (Kohlbacher, 2006). The transcripts were paraphrased, abstracted, and reduced to preserve essential content, using as a coding system the scaling-up categories and resources requirements from ScalA tool.

For the quantitative analysis, average scores of the compliance of 59 scaling-up factors were calculated per system and per region to identify individual hindering and fostering factors. Average scores less than 1 denote hindering factors and average scores greater than 1 denote fostering factors. Furthermore, scores for the 11 different resource requirements were averaged by system and by region, facilitating the visualization of the most significant resource requirements affecting SPS and CAFS scaling-up in each region. The scaling-up percentage deviations of each interview and focus group (both total and by scaling-up category) were automatically calculated by the ScalA tool. Finally, we calculated the averages of these percentage deviations by system, disaggregating them by region to allow for comparisons of the scaling-up categories between regions. The higher the percentage deviation of the category, the more it hinders scaling-up.

## Results

### Hindering and fostering factors to scale up SPS and CAFS

When assessing scaling-up potential through the 59 ScalA factors, some differences and commonalities between AFS and between regions emerge. These are presented here by scaling-up category; additional details are shown in Supplementary file 1.

#### AFS attributes

Regarding affordability, average scores show two hindering factors when scaling-up both AFS: farmers lack sufficient financial means and have difficulties accessing required external inputs. In terms of AFS complexity, results show that farmers need regular trainings to implement SPS in both regions and CAFS in Caquetá. According to CAFS experts in Cesar, these systems and related practices are known to farmers since they have traditionally managed permanent crops alongside agroforestry. Another factor potentially hindering AFS scaling-up is the level of social organization. This is why some interviewees agree that it is necessary to strengthen local social organizations when implementing CAFS projects. Finally, two factors potentially hinder SPS in both regions, albeit more critically in Cesar: farmers cannot quickly reap benefits and perceive a higher economic risk versus the alternatives. Regarding fostering factors, farmers tend to have easy access to organic inputs since their own organic waste can produce fertilizers; however, respondents perceive greater difficulty in accessing seeds and plant material, especially for implementing SPS in Cesar. All respondents also agree on: AFS can be tried out on small plots where benefits can easily be observed; AFS fit into existing production systems, thus increasing its long-term efficiency; and farmer autonomy and independence can thrive with AFS. SPS and CAFS also offer the potential for value-adding in order to increase benefits, but required structures have to be strengthened, mainly in Caquetá.

#### Capacity of implementing organizations

Interviewees generally perceive that a clear and transparent structure of the implementing organization is key for scaling-up. Especially in Caquetá, respondents appreciate the management and technical staff. Interviewed organizations do not just have strong leadership with a good reputation among beneficiaries, but they also have branch offices or a regional network comprising like-minded organizations. Organizations also have access to well-established networks of donors, policymakers, researchers, and private sector institutions, mainly in Caquetá due to its ecological importance. However, AFS promotion in both regions is restricted by the limited availability of technical staff.

#### AFS scaling-up strategies attributes

Generally, organizations have a clear definition of the scaling-up objective, a clear strategy for this objective, along with a well-established documentation, monitoring, and evaluation system. To promote AFS, they use effective dissemination channels, high-quality partnerships with farmers, and minimal incentives to introduce AFS. Some deficiencies are perceived by respondents regarding the engagement of their organizations in strengthening local organizations that support SPS promotion and implementation in both regions.

#### National-level political/institutional framework

Respondents perceive sociopolitical tensions that could hinder the scaling-up of SPS in both regions and CAFS in Caquetá. Moreover, the government still fails to effectively integrate AFS in formal curricula or in research and extension programs; this greatly hinders SPS scaling-up in both regions as they are not the traditional cattle ranching systems. Most respondents indicate that the governmental administrative system tries to support AFS scaling-up activities, but its agriculture and development agencies have limited efficiency or are physically absent. Finally, the governance system must be improved while scaling-up CAFS and SPS in both regions because there are decentralized structures that allow local solutions but lack effectiveness.

#### Local institutional setting

Generally, respondents report that local government development plans support AFS-related activities but lack financial and human capacities. Similarly, local associations are willing to support AFS activities, but they lack effectiveness and require strengthening. Experts also perceive unclear structures for land use and access due to informal land rights that might hinder AFS scaling-up, especially in Cesar.

#### Local/regional economic conditions

There are clearly more limitations for scaling-up SPS in both regions and CAFS in Cesar, since the predictability and attractiveness of market prices for cacao, fruits, milk, and meat produced under AFS are not advantageous compared to conventionally produced. However, while CAFS markets in Caquetá seem to guarantee better prices, their predictability and stability remain uncertain. Although there are multinational, national, and local food processing companies, who could benefit economically from AFS-derived agricultural products, their interest and support are uncertain. General infrastructural necessities, including access to roads, electricity, and water, are lacking in both regions. Availability of processing facilities is a hindering factor for both farming systems in both regions since farmers usually sell cacao and cattle products without any value-added transformation. Finally, AFS promoting organizations can help producers improve productivity, but accessing certification structures remains difficult.

#### Community attitudes toward AFS

AFS scaling-up could be fostered in both regions according to the respondents since not only does the majority of community members welcome AFS, but leaders also generally accept it. However, the limited number of young farmers interested in AFS and the limited number of community members engaged in entrepreneurial activities are concerning. Finally, although farmers are willing to actively participate in AFS project activities, concerns regarding cost/labor-sharing and the needed human capacities remain.

### Resource requirements influencing SPS and CAFS scaling-up

**Fig. 1:**
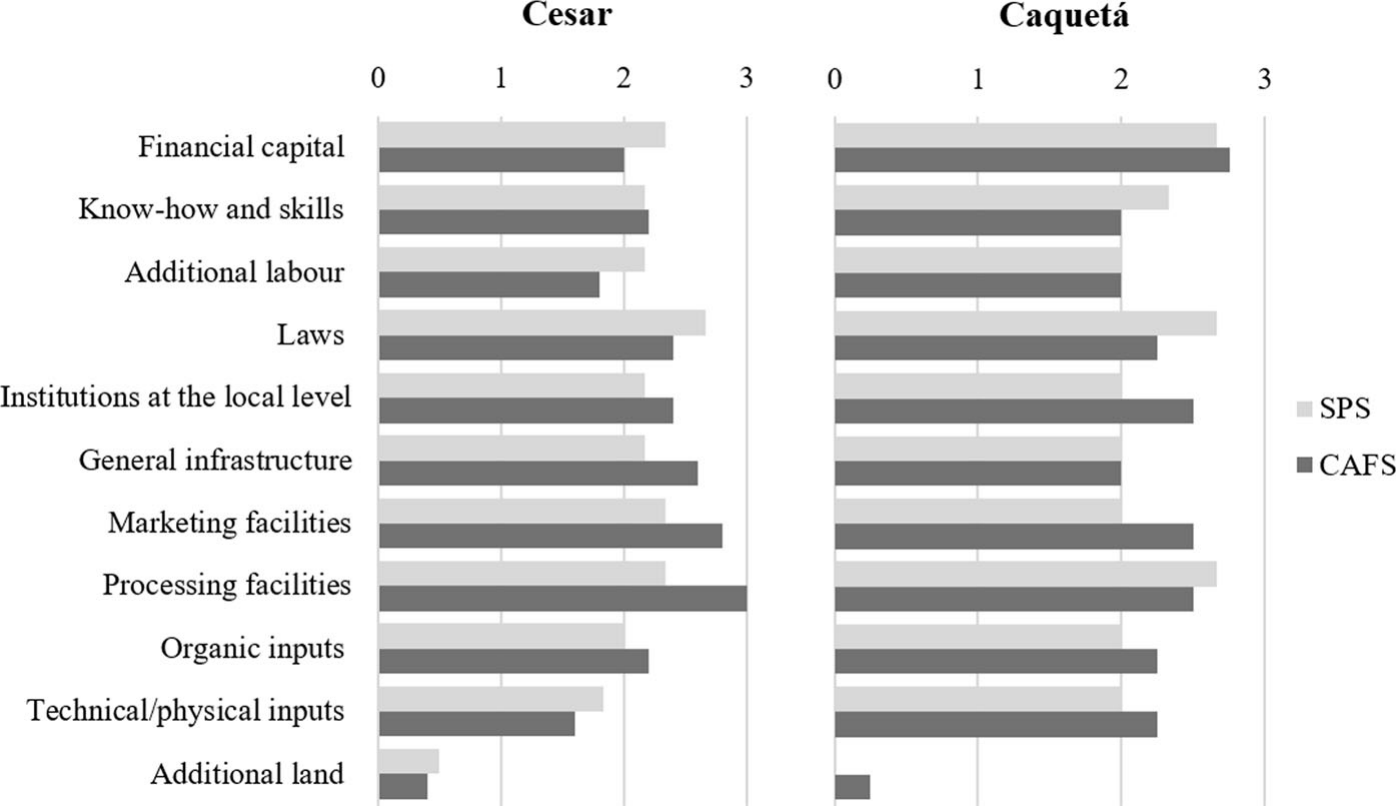
Overview of financial, human, institutional, infrastructure, and agricultural input resources requirements for implementing Silvopastoral Systems (SPS) and Cacao Agroforestry Systems (CAFS) in Cesar and Caquetá. Experts scoring from 0 to 3: 0 = 'not relevant'; 1 = 'low'; 2 = 'medium'; 3 = 'high' (n_Cesar_ = 11, n_Caquetá_ = 7)

Figure 1 presents an overview of resources requirements and expert scores regarding perceived relevance for implementing SPS and CAFS.

Respondents perceive high financial capital is required to implement SPS and CAFS, in Caquetá slightly more than in Cesar. Know-how and skills are also perceived as medium or highly demanding for both systems, since both incorporate more elements into the agricultural system, thus requiring proper management. One interviewee notes this is relevant for both farmers and the staff advising them. These systems imply additional labor, perceived as medium or high for SPS; low or medium for CAFS. Some experts agree on the demand for financial capital, know-how, and additional labor depend on AFS complexity.

The need for specific agricultural and natural resource management laws as well as local institutions to scale up AFS is perceived as medium or high. The former is more required by SPS and the latter is more needed by CAFS in both regions. General infrastructure is perceived as a medium demanding resource for both systems in both regions. However, CAFS experts perceive that there is a slightly higher demand for infrastructure in Cesar since cacao farmers require a water distribution system. Similarly, demand for marketing facilities is generally perceived by experts as medium, since there are already local facilities where agricultural products of both systems could be sold. Processing facilities seem to constrain SPS and CAFS scaling-up in both regions since both require them at a medium or high level. For example, experts indicate that cacao producers in both regions typically lack adequate facilities for post-harvest processes.

In terms of external inputs, organic inputs are mostly assessed as a medium requirement. Technical and physical inputs are mostly assessed as a low or medium requirement in Cesar, but medium and high in Caquetá. Although these systems aim to reduce external fertilizers use, Caquetá's soils require them more than Cesar's soils. The demand for additional land is scored as not relevant or with a low relevance for both systems. Cattle farms are considered large enough in both regions, with SPS implementation implying less land due to its sustainable intensification practices. Cacao farms in Cesar are traditionally family farms unable to enlarge due to labor limitations.

### SPS and CAFS scaling-up potential

**Fig. 2:**
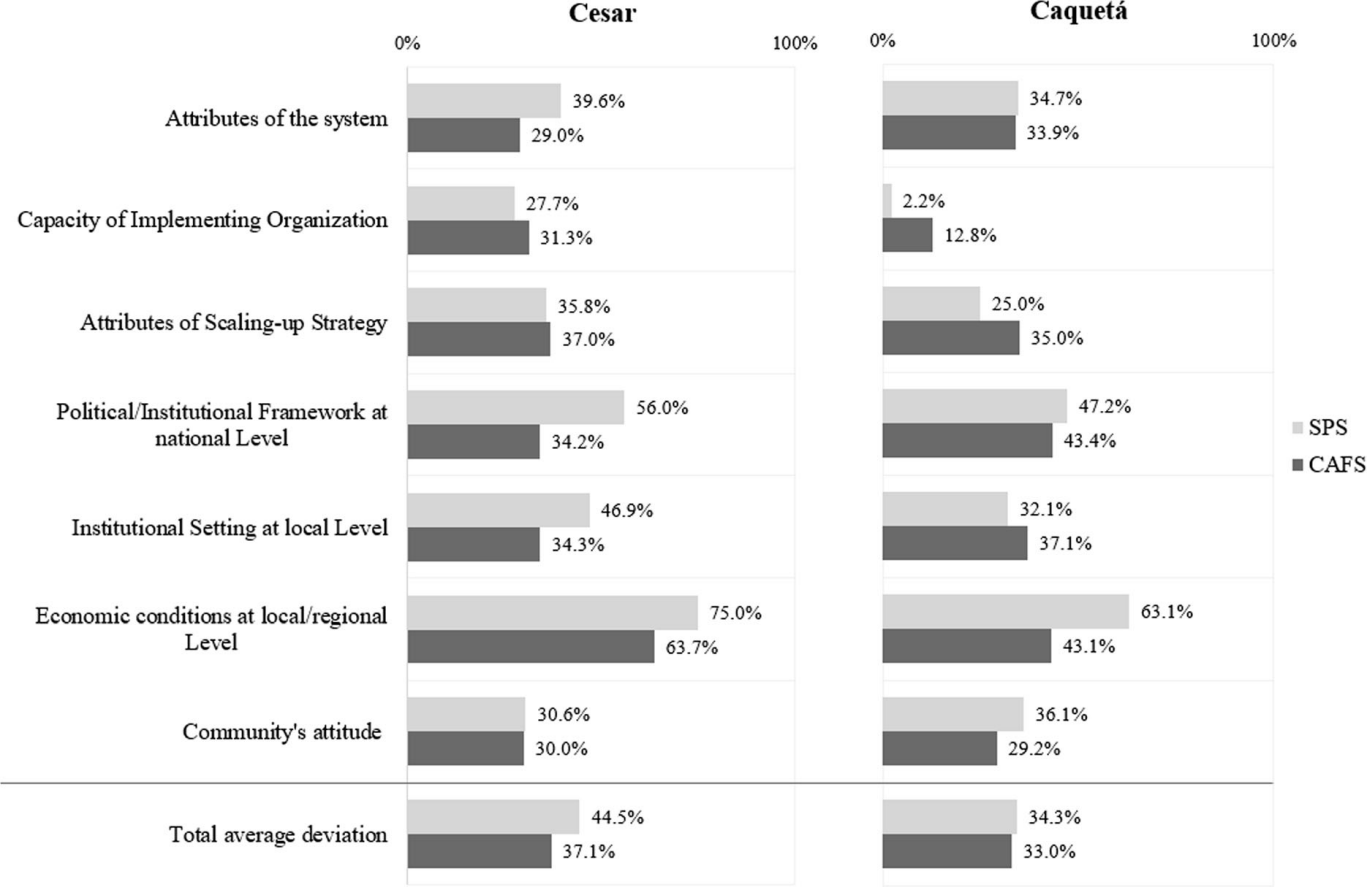
Overview of 7 scaling-up categories of factors and average percentage deviation from the optimal scaling situation based on experts' scoring of resource requirement and rating of scaling-up factors. Lower percentages denote smaller deviations from the optimal situation, that is, categories that hinder less SPS/CAFS scaling-up. Higher percentages denote higher deviations from the optimal situation, that is, categories that hinder more SPS/CAFS scaling-up. The last bars show the total average deviation; (n_Cesar_ = 11, n_Caquetá_ = 7)

Regarding the total scaling-up potential for each system (Fig. 2), results show differences between AFS and regions, thus supporting the need to create context-specific interventions. Total average deviations from the optimal situation indicate that the potential for scaling-up SPS and CAFS is higher in Caquetá than in Cesar. This relates mainly to the capacities of implementing organizations in Caquetá. Of these farming systems, the scaling-up potential is slightly higher for CAFS than SPS, clearly in Cesar. This is an expected result since cacao farming systems in Colombia have traditionally been managed as AFS. There, the attributes of the system, the political/institutional settings, the economic conditions, and community’s attitudes pushed CAFS to be preferred over SPS, even though SPS is more favorably assessed with respect to institutional capacities and scaling-up strategies. In Caquetá, the results are similar, but the local institutional setting slightly favors scalability of SPS. The most hindering category for SPS and CAFS scaling-up in both regions refers to the local and regional economic conditions while the least hindering category has to do with the capacity of implementing organizations.

## Discussion

Our hypothesis was confirmed through this study: economic conditions are the greatest hindrance to SPS and CAFS scaling-up in both regions. In this sense, implementing organizations should encourage SPS and CAFS market-oriented interventions (Pandit et al., 2019), where producers associations are strengthened and connected with the private sector to establish marketing platforms, especially for meat, milk, and dried cacao. For value-added products, like cheese derived from SPS, the focus should be on supporting and strengthening local value chains, as Reij and Garrity (2016) suggest, through the improvement and formalization of storage and processing facilities. Other strategy should be technically and economically supporting farmers on getting certifications (e.g., organic) to obtain premium prices (Andres et al. 2016; Guteta and Abegaz 2016; Rosati et al. 2021).

AFS scaling-up is also influenced by the social, institutional, and biophysical conditions of the regions as well as the resources required to implement and sustain these systems. Like other studies (Mahecha 2003; Calle et al. 2009; Chitakira and Torquebiau 2010), we find the assessed AFS require medium to high financial capital for the establishment and maintenance, something farmers typically lack. To overcome this hindrance, low-cost SPS arrangements using natural regeneration of native trees could be promoted in both regions. Natural regeneration costs nothing in some cases because new trees grow without nurturing or protection (Reij and Garrity 2016), and it provides an opportunity to restore degraded lands (Mauricio et al. 2018). In the case of CAFS, organic management could also be encouraged in both regions to decrease financial capital required and increase farm income since it does not seem to negatively affect cacao yields or incidence of pests and diseases (Padmavathy and Poyyamoli 2013; Schneider et al. 2017; Armengot et al. 2020). Another strategy could be focused on encouraging SPS and CAFS establishment by providing incentives, like external agricultural inputs (Chitakira and Torquebiau 2010). However, these incentives must be well planned, adapted, and monitored because they could affect the intrinsic motivations of farmers to implement these sustainable systems. For example, Kakhobwe et al. (2016) state that the long-term provision of agricultural inputs could negatively affect AFS scaling-up by farmers.

Farmers also need greater technical knowledge and skills when managing SPS and CAFS. As this hinders AFS scaling-up, more effective extension services must be provided. Here, the active involvement and integration of farmers with extension workers and researchers through on-farm demonstration plots is key: farmers will not just help to setup, manage, and monitor these plots, but will also support AFS scaling-up after observing its benefits (Bertin et al. 2014; Jagoret et al. 2014; Kakhobwe et al. 2016; Guteta and Abegaz 2016). Farmers input is crucial in planning where and which tree and shrubs species to plant, as they know which are complementary to their crops and cattle (Jose et al. 2019; Baig et al. 2020). Promoting farmer participation in research and extension seems viable, as scaling-up factors related to community attitudes toward AFS are positively assessed. Further, training AFS staff is essential to enhance its scaling-up (e.g., Chitakira and Torquebiau 2010; Landicho et al. 2009).

Although the literature suggest that the integration of trees, crops, and/or livestock through AFS can contribute toward resource use efficiency and sustainable livelihoods, these systems must be well integrated considering the local biophysical and socioeconomic conditions. For example, SPS and CAFS scaling-up potential in both regions decreases due to perceptions of higher economic risk. For SPS, it might be associated with biophysical conditions of the regions (e.g., long droughts in Cesar, floods in Caquetá) that hamper the survival and growth of plants when establishing them. For CAFS, it might be related to incipient timber markets, as the producer’s ability to harvest and sell it is limited due to governmental policies (Abbott et al. 2018).

Although specific agricultural and natural resource management laws are needed for AFS scaling-up, no established framework or specific law for AFS exists in Colombia, just as in other countries (Macke et al. 2021). However, as Callo-Concha et al. (2017) point out, given its diverse practices, AFS is affected by multiple policies. Thus, a legislative framework that comprehensively supports AFS is a policy challenge to address at the national level.

Franzel et al. (2004) identify the building of local institution capacity as a key factor for scaling-up AFS. This is consistent with our results, which indicate strengthened local social organizations will effectively underpin scaling-up AFS. These organizations will not only sustain AFS in the long term but also facilitate the understanding and mitigation of unintended effects (Castro-Nunez et al., 2021). However, the scaling-up assessment shows that local governments and local-based organizations in the regions lack institutional capacities, while AFS supportive organizations often lack strategies to strengthen local-based organizations, especially in Cesar. Here, establishing strategic public–private partnerships could support AFS scaling-up, as highlighted in existing studies (Chavan et al. 2015; Macke et al. 2021).

## Conclusions

This comparative approach shows how context-specific AFS interventions must be planned in order to increase their effectiveness and their subsequent scaling-up. However, it also identifies some commonalities relevant to other regions and farming systems. To scale up AFS, enabling economic conditions that protect farmers against market risks, either by seeking more favorable prices or by strengthening local value chains to generate greater added value, is critical. Further, AFS projects should incentivize farmers to establish AFS while promoting low-cost arrangements based on local farmer-centered research and planning, facilitating compatible and synergetic relationships between AFS elements. Considering the complexity of AFS implementation and management, institutions supporting AFS should actively bring farmers, extension workers, and agricultural researchers together. Strategic partnerships between public, private, and local organizations should be established to facilitate and guarantee AFS sustainability.

However, study limitations must be considered when observing its results. First, broad definitions of SPS and CAFS are considered; however, particular arrangements of these systems may have different scaling-up implications. Second, the case study approach followed, focusing on two AFS and two regions, may limit the generalizability of the findings. Third, although the most experienced and knowledgeable respondents of AFS-related institutions were interviewed, including some farmers, considering a broader set of perspectives would provide more insights into AFS scaling-up hindrances.

## Supplementary Information

Below is the link to the electronic supplementary material.

Supplementary file1 (PDF 67 kb)

## Data Availability

The data that support the findings of this study are available on request from the corresponding author, Tatiana Rodríguez. The data are not publicly available because it contains information that could compromise the privacy of research participants.
