# Supplementary material for: Analyzing influencing factors to scale up agroforestry systems in Colombia: A comparative ex-ante assessment of cacao farming and cattle ranching in two regions
Source: Agrofor Syst. 2022 Jan 18;96(2):435–46. doi: 10.1007/s10457-022-00730-1 (PMC8765677; doi:10.1007/s10457-022-00730-1)
Supplement: Supplementary file 1 — Supplementary file1 (PDF 67 kb) [file 10457_2022_730_MOESM1_ESM.pdf]

Supplementary file 1. Scaling-up categories of factors and average scores

Category	Factor	Average score			
		SPS		CAFS	
		Cesar	Caquetá	Cesar	Caquetá
AFS attributes	1: Is the practice affordable by farmers or can the target group afford the cost of the AFS?	0.67	0.67	0.60	0.25
	2: Is the knowledge required for successful implementation available/is the AFS already known to the farmers?	1.00	1.33	1.00	0.75
	3: Is the AFS easy to understand and implement?	0.50	0.33	1.40	0.75
	4: Is the AFS initially adapted to labor endowment of farms?	1.17	1.00	1.00	1.25
	5: Is the level of social organization that is required for the implementation of the AFS available within the target community?	0.50	1.00	1.00	1.00
	6: Are the organic inputs required for successful implementation of the good practice initially available to farmers?	1.00	1.33	1.20	1.25
	7: Are the technical inputs required by the AFS initially available to farmers?	0.67	0.67	0.40	0.75
	8: Can the AFS initially be implemented on existing farms without requiring additional land OR is the required additional land available to the farmers?	1.67	2.00	2.00	1.50
	9: Are the benefits of the AFS easily observed by farmers?	1.67	2.00	1.80	2.00
	10: Can farmers quickly reap benefits from the AFS?	0.67	1.00	1.20	1.50
	11: Is the economic risk for farmers comparatively low?	0.83	1.00	1.40	1.50
	12: Is it possible for farmers to try out and verify the effects of the AFS by testing it at small scale?	1.50	1.67	2.00	1.75
	13: Is the implementation of the AFS flexible, i.e. can it be easily modified by other farmers to suit different ecologic and socio-economic circumstances and needs within and outside the target area?	1.83	2.00	1.80	2.00
	14: Does the AFS fit into the existing farming AFS and does it improve its efficiency?	1.67	2.00	1.80	1.25
	15: Does the AFS contribute to the farmer's autonomy, prestige, and independence?	1.33	1.33	2.00	1.50
	16: Does the AFS not increase pressure on natural resources such as water and land?	2.00	2.00	1.60	2.00
	17: Does the AFS offer potential for value adding in order to increase benefits?	1.33	1.00	1.40	1.00
Capacities of implementing organizations	1: Does the organization have a well-designed overall concept, e.g. a binding rule system and a transparent structure?	1.00	2.00	1.60	2.00
	2: Does the organization have well-educated technical and management staff?	1.17	2.00	1.00	2.00
	3: Does the organization have strong leadership with a good reputation among the beneficiaries?	1.50	2.00	1.80	1.75
	4: Does the organization have access to an already existing structure of branch offices or other organizations based in the target area?	1.67	2.00	1.20	1.25
	5: Does the organization have experience with the target group?	1.50	2.00	1.00	1.75
	6: Does the organization have access to well-established networks of donors, policy-makers, researchers, and the private sector?	1.50	1.67	1.40	1.75
	7: Is the staff trained to ensure participatory project selection and planning using cultural sensitive approaches?	1.33	2.00	1.00	2.00
	8: Does the organization have access to basic assets, like power, and to relevant communication systems?	1.83	2.00	1.80	2.00

Category	Factor	Average score			
		SPS		CAFS	
		Cesar	Caquetá	Cesar	Caquetá
Attributes of scaling-up strategies	1: Is the objective of scaling-up clearly defined?	1.17	1.33	1.60	1.33
	2: Does the organization have a clear strategy to reach the objective by defining the type, sequencing and means employed for scaling-up their activities?	1.17	1.33	1.20	1.50
	3: Does the organization have a well-established and effective documentation, monitoring, and evaluation system?	1.17	1.67	1.00	1.50
	4: Does the implementing agency use already existing information channels?	1.50	2.00	1.20	1.25
	5: Does the implementing agency use effective and efficient dissemination channels to promote/disseminate the AFS?	1.33	2.00	1.40	1.50
	6: Does the organization use efficient means of spread of information adequate to each type of target audience?	1.50	1.67	1.20	1.50
	7: Does the organization have high-quality partnerships with farmers, with feedback mechanisms and exchange of experience?	1.17	1.33	1.20	1.00
	8: Is the organization engaged in capacity building, and the implementation of sustainable supportive organizations at the local level?	1.00	1.33	0.80	1.50
	9: Does the implementing organization promote the AFS through mass media, such as radio, TV, and newspapers?	1.17	1.00	1.20	0.75
	10: Does the organization use only minimal incentives to introduce project activities?	1.67	1.33	1.80	1.50
National level political/institutional framework	1: Is there political stability in the area/country?	0.83	0.67	2.00	0.75
	2: Does the government promote a supportive land, water and agricultural policy that facilitates the introduction/dissemination of project activities among the target group/within the target area?	1.00	1.00	1.00	1.25
	3: Does the government support project activities/AFS through research and extension?	0.83	1.33	0.80	1.25
	4: Does the government have an efficient administration system that facilitates (or does not hinder) scaling-up activities?	1.00	1.00	1.00	1.00
	5: Is the governance system structured in an adequate, decentralized way?	0.67	1.00	1.20	1.00
	6: Is the situation of civil society conducive to scaling-up of project activities at the local and regional/national levels?	1.00	1.33	1.80	1.50
Local institutional setting	1: Is there a functioning local level governance structure that can act as cooperation partners for scaling-up?	1.17	1.00	1.20	1.00
	2: Are there already local organizations that can be used during the process of scaling-up to facilitate the dissemination of project activities/the AFS?	1.17	1.00	1.40	1.00
	3: Are there local rules that support or do not hamper scaling-up of project activities/the AFS?	1.00	1.67	1.40	1.75
	4: Do the usage/access rights to land support, or do not hamper, the introduction of project activities/the AFS?	0.83	2.00	0.80	1.50
	5: Does the spatial distribution of households not hamper project activities?	1.17	1.00	1.80	1.00

Category	Factor	Average score			
		SPS		CAFS	
		Cesar	Caquetá	Cesar	Caquetá
Local/regional economic conditions	1: Is there a stable market that can absorb the AFS products?	0.33	0.67	0.60	1.50
	2: Are markets and marketing facilities easily accessible by farmers?	0.67	1.33	0.60	1.75
	3: Is the market price for the produce of the AFS predictable and attractive?	0.33	0.67	0.60	1.25
	4: Is there interest/support for the spread of the AFS by other economic actors?	0.67	1.00	0.80	0.75
	5: Is the necessary infrastructure, like access to roads, irrigation, electricity, and tap water, available to the target group?	0.17	0.33	0.80	1.00
	6: Are processing facilities available to the target group?	0.33	0.33	0.60	0.00
	7: Are there mechanisms that will enable farmers to eventually meet particular standards required by regional/national/international markets?	1.00	1.00	1.00	1.50
Community attitudes toward project activities	1: Are project activities/the AFS welcomed by the majority of the community?	1.50	1.67	1.60	1.50
	2: Are project activities/the AFS accepted by village leaders?	1.50	2.00	1.60	2.00
	3: Are project activities/the AFS welcomed by young farmers?	1.50	1.00	1.20	1.00
	4: Is the target group willing and able to actively participate and cooperate in project activities/the introduction of the AFS?	1.33	1.00	1.40	1.50
	5: Is individual engagement in project activities socially accepted?	1.50	1.00	1.80	1.50
	6: Is there entrepreneurial behavior within the community?	1.00	1.00	0.80	1.00

The highlighted dark grey cells denote the hindering factors and the highlighted light grey cells denote the fostering factors.
